# New Techniques for the Study of Neural Tube Defects

**DOI:** 10.4172/2379-1764.1000157

**Published:** 2015-12-26

**Authors:** Yunping Lei, Richard H Finnell

**Affiliations:** 1 Dell Pediatric Research Institute, Department of Nutritional Sciences, The University of Texas at Austin, Austin, Texas; 2 Dell Children’s Medical Center, Austin, Texas

## Abstract

Neural tube defects (NTDs) are among the most common complex congenital malformations observed in newborns. When the neural tube fails to close completely, severe malformations of the brain and/or spinal cord and subsequent neurologic impairment occurs. It is widely believed that nutritional, environmental and genetic interactions contribute to NTDs. It is well established that low folate levels during pregnancy increases a mother’s risk of having pregnancy complicated by an NTD, and providing periconceptional folate supplementation reduces this risk. The underlying genetic mechanisms of NTDs are still unclear. We review the many new approaches to better understand the etiology, especially the genetic etiology, underlying this family of birth defects.

Neural tube defects (NTDs) are characterized by a failure of neural tube closure during early embryonic development. The most frequent types of NTDs are spina bifida, which are defects of low spinal closure below the level of T12, and anencephaly, which results from incomplete closure of cranial neural tube. Failure of the neural folds to elevate results in the entire neural tube remaining open is referred to as craniorachischisis. The worldwide prevalence of NTDs is 0.5–1 per 1000 newborns, with variations among different populations [1]. The etiology of NTDs is complex, including both genetic and environment factors. In mice, so far there are more than 300 genes were linked to NTDs [2]. However, no causative mutations have been identified in humans to date. One possible reason is that there are very few large, multigenerational families that could be used to identify causative NTD genes using linkage mapping. Other obstacles to identifying NTD causative genes using mouse models is that most of these gene knockout models do not express an NTD phenotype as heterozygotes, yet the homozygous embryos most often suffer from *in utero* lethality.

In thinking about the genetic basis of NTDs, many investigators consider the notion that multiple, combined heterozygous variants in same gene, same pathway, genetic or physical interaction partner, work together to produce the NTD phenotype in humans. These combined functional variants could be inherited or result from germline *de novo* and/or somatic *de novo* mutations. However, it has been very difficult to directly test this hypothesis, due to the limitations of our existing genome editing technologies. Recently, the development of next generation sequencing (NGS) techniques [3] and clustered regularly interspaced short palindromic repeats (CRISPR)-Cas9 genetic editing technique [4] provide an excellent opportunity to test this hypothesis.

Next generation sequencing (NGS) also called (high throughput) massive parallel sequencing including NGS-whole exome sequencing (NGS-WES), NGS-whole genome sequencing (NGS-WGS), and NGS-target enrichment sequencing. Compared with first generation (Sanger) sequencing, the newer approaches can generate large amounts of sequencing data in a short time at a reasonably low cost. For example, the human genome sequencing project took 13 years and cost over $3 billion dollars. Using the latest NGS equipment (eg. Illumina HiSeq 4000), sequencing a whole human genome can be completed in a week at a cost approaching $1,000. Since more than 300 genes have been reported to be involved in murine neural tube closure, it is highly likely that even more genes that contribute to the expression of human NTDs will be discovered. We believe that one approach to identifying new candidate NTDs genes in humans, is to appropriate the NGS-WES and NGS-WGS methodologies/strategies that are currently being successfully used for identifying risk genes in autism spectrum disorder [5], a multifactorial disease similar to NTDs. For human NTDs, it is also assumed that multiple related (eg. in a pathway) functional variants combined can be the underlying genetic etiology of some cases. Thus far, millions of genetic variants have been identified; therefore, the potential combinations of multiple variants could be in the billions or trillions. To test whether combined rare variants in a pathway are human NTD genetic risk factors, scientists need to sequence thousands of NTD cases for all the known candidate pathway genes. The NGS-target enrichment sequencing technique is perfect for this purpose. Currently, there are three types of target capture/enrichment methods: multiple- PCR based method capture, hybrid capture (on-array or in-solution) and molecular inversion probes (MIP) capture [6]. Each method has its advantages and disadvantages. MIP has been successfully used for autism risk genes validation in a large sample size due to its low cost, ease of use, and template saving advantages [7]. We believe that this technique has the potential successfully enhance our understanding of NTDs risk genes/pathways by performing validation studies on large NTD cohorts.

The functional characterization of identified variants is important for judging whether the detected combination of variants is causative or not. The most direct way is to “knock-in” the variants into mice and then determine whether these variants induce an NTD phenotype, for murine neural tube closure shares much in common with human neural tube closure, perhaps more closely than neural tube closure in zebrafish or in *Xenopus*. The latest genetic editing technique, CRISPR-Cas9, can also be utilized for functional studies of identified gene variants. Compared with CRISPR-Cas9, traditional genetic editing techniques including homologous recombination, zinc finger nuclease, and transcription activator-like effector nuclease are laborious, expensive and time consuming. It usually takes 12–18 months to make a knock in mouse line. CRISPR-Cas9 technology can easily generate genetically modified mice in one month [8,9]. Recently, Zhong and colleagues used CRISPR-Cas9 technology to edit androgenetic haploid embryonic stem cells (AG-haESCs), and successfully injected the genetically modified AG-haESCs into MII oocytes that developed into liveborn mouse pups [10]. The combination of these two new techniques working together should vastly improve our ability to perform the functional screening of human NTDs variants quickly and efficiently. Most of the identified variants in human NTDs are heterozygous mutations, such as the functional variants identified in planar cell polarity (PCP) pathway genes including: VANGL2 [11], VANGL1 [12], CELSR1 [13], SCRIB [14], LRP6 [15], FZD6 [16] and DISHEVELLED2 [17]. PCP single gene knock out heterozygotes do not produce an NTD phenotype in mice, while PCP double heterozygotes do result in multiple types of NTDs in mice [18]. Based on the mouse data, it is easy to hypothesize that a double loss of functional variants in PCP genes could also cause NTDs in human. To test this hypothesis, we need to screen a large number (thousands) of NTD samples for PCP genes using one of the aforementioned NGS-target enrichment techniques in order to identify those variants combinations. AG-haESCs could be used to make genetic “knock in” mice that contain the same variants combinations as observed in human NTD cases. Whether or not these “knock in” mice present with NTDs phenotype will be the hallmark by which to judge the NTD causality of these variant combinations. The same methods could also be used for other candidate NTD gene pathways, such as the sonic hedgehog pathway, one-carbon metabolism pathway, cilia pathway, cell apoptosis pathway, and more.

In summary, NGS, CRISPR-Cas9 and AG-haESCs are the state-of-art techniques and tools whose utilization will advance our understanding of the etiology of NTDs.
